# The Study on Cervical Cancer Burden in 127 Countries and Its Socioeconomic Influence Factors

**DOI:** 10.1007/s44197-022-00081-1

**Published:** 2022-12-21

**Authors:** Tingting Xu, Xueling Yang, Xiaoning He, Jing Wu

**Affiliations:** 1 School of Pharmaceutical Science and Technology, Tianjin University, Tianjin, 300072 China; 2 Tianjin Medical University Cancer Institute and Hospital, Tianjin, 300060 China

**Keywords:** Cervical cancer burden, Socioeconomic factor, HDI, Cancer control strategy, Policy effectiveness

## Abstract

**Objectives:**

To explore the relationship between cervical cancer burden and HDI and other socioeconomic influence factors in 127 countries.

**Methods:**

Dividing 127 countries into low-, medium-, high-, ultrahigh-HDI groups, and using statistical method to compare the prevalence trend of cervical cancer in different HDI country groups. Then selecting mortality-to-incidence ratio (MIR) to measure the cancer burden as the dependent variable, HDI and other socioeconomic factors selected from 2020 Human Development Report as independent variables, and using multi-regression model to analyze the correlation between variables.

**Results:**

Countries with higher HDI were found to have lower prevalence and mortality of cervical cancer, and vice versa. Besides that, air and water pollution, government-coordinated spending, and the intimate partner of 15–49-year-old women and girls have positive correlation impact on cervical cancer burden.

**Discussion:**

The cancer prevention and control policies in countries with high HDI have achieved relatively ideal implementation effects. Countries with relatively backward social and economic development level, cancer prevention and control policies had little effect, such as lower HPV vaccination coverage, poor regional health resource allocation, and week public education awareness. Therefore, cervical cancer control plan must be integrated into national strategies and implemented in people-oriented comprehensive health services.

## Introduction

Cervical cancer is a malignant tumor caused by human papilloma virus (HPV) infection, which has become the killer of malignant tumors in the female reproductive system [1]. 2020 Global Cancer Report statistics showed that there were 530,000 new cervical cancer cases and 250,000 deaths per year worldwide, of which 85% occurred in low- and middle-income countries [2]. Due to the high prevalence of HPV vaccination and screening in western developed countries, the incidence of cervical cancer in developed countries shows a slow downward trend. However, the prevalence of cervical cancer in low- and middle-income developing countries is more serious due to the uneven allocation of health resources, single health financing channels, lack of policy support and many other reasons [3].

The Human Development Index (HDI) is a statistic developed and compiled by the United Nations to measure various countries’ levels of social and economic development. It is composed of four principal areas of interest: mean years of schooling, expected years of schooling, life expectancy at birth, and gross national income (GNI) per capita [4]. This index is a tool used to follow changes in development levels over time and compare the development levels of different countries [5]. To realize why cervical cancer burden is heavier in low- and middle-income countries, this research will explore the relationship between cervical cancer burden and HDI and other socioeconomic impact factors. The study will choose 127 countries worldwide and divide them into low-, medium-, high-, ultrahigh-HDI groups, use statistical method to compare the epidemiology trend of cervical cancer in different HDI country groups. Then choosing mortality-to-incidence ratio (MIR) to measure the cancer burden as the dependent variable, HDI and other socioeconomic factors selected from 2020 Human Development Report as independent variables, use multi-regression model to analyze the correlation between variables. Based on the quantitative analysis, this research will further discuss the cervical cancer control strategy and its implementation in different HDI country groups, and provide political suggestions.

## Methods

### Data Source

The epidemiological data including world age-standardized incidence and mortality, and disability-adjusted life years (DALY) are from the WHO Cancer Tomorrow 2020 and Global Burden of Disease Database (GBD). The data associated with socioeconomic factors such as HDI index are from World Health Statistics 2020 and Human Development Report 2020.

### Analysis Method

The study will use descriptive statistical analysis to compare the differences in the world age-standardized incidence of cervical cancer (hereinafter referred to as standardized incidence), world age-standardized mortality (hereinafter referred to as standardized mortality) and disability-adjusted life years of cervical cancer at the median level and the upper and lower quartiles of each HDI level group. The Kruskal–Wallis *H* non-parametric test was used to compare the disease prevalence differences among different HDI country groups.

Mortality-to-incidence ratio (MIR) is recognized as an effective predictor of disease screening, treatment and prognosis, and can also be used to reflect cancer burden. This research chose mortality-to-incidence ratio (MIR) as the dependent variable to measure the cervical cancer burden [6].

$$\mathrm{MIR}=\frac{\mathrm{Mortality }}{\mathrm{Incidence}}\times 100\%.$$

The study selected the factors including total population, proportion of ever-partnered women and girls aged 15–49 years subjected to physical and/or sexual violence by a current or former intimate partner in the previous 12 months (%), density of pharmacists (per 10 000 population), density of medical doctors (per 10 000 population), average of 13 International Health Regulations core capacity scores, amount of water- and sanitation-related official development assistance that is part of a government-coordinated spending plan (current US$ millions), proportion of population using safely managed sanitation services (%), annual mean concentrations of fine particulate matter (PM2.5) in urban areas (µg/m3) from 2020 World Health Statistics, and adjusted HDI index from 2020 Human Development Report as the independent variables. Multiple linear regression models were used to analyze the impact of HDI and other socioeconomic factors on the cervical cancer burden.

## Results

### Cervical Cancer Prevalence in Different HDI Level Country Groups

In Table 1, 127 countries were classified into four groups: ultrahigh HDI, high HDI, medium HDI, and low HDI levels. Graphpad Prism 8.3.0 software was used in this section to perform descriptive statistical analysis of cervical cancer incidence, mortality and DALYs prevalence, including median and 95% confidence interval (95% CI), upper quartile, and lower quartile, in different HDI levels (Table 2). The Violin Plot was also drawn to show the distribution and frequency of the data (Graph 1–3).

**Table 1:** Country classification list in different HDI levels

HDI level	Country list
Ultrahigh	Estonia, panama, Belarus, Poland, the Russian federation, Costa Rica, Kazakhstan, Montenegro, Czech republic, Latvia, Lithuania, Malaysia, Serbia, Hungary, Slovakia, Brunei, Uruguay, Chile, the united Arab emirates, Oman, South Korea, Austria, Bahrain, Qatar, Kuwait, Croatia, Luxembourg, Turkey, Portugal, Cyprus, Saudi Arabia, Turkey, Greece, Singapore, New Zealand, Slovenia, Italy, Barbados, Bulgaria, Romania
High	Azerbaijan, north of the republic of Macedonia, Bosnia and Herzegovina, Ecuador, cape Verde, Cuba, Gabon, Mongolia, Samoa, Thailand, Peru, Moldova, Ukraine, Uzbekistan, Turkmenistan, Armenia, Algeria, Indonesia, Vietnam, China, Albania, Egypt, Pakistan, the Philippines, Lebanon, Libya, Maldives, Sri Lanka, Tunisia, Iran, Israel, Jordan, Bolivia, Dominica, Fiji, South Africa, Suriname, Trinidad and Tobago, Venezuela, Jamaica
Medium	East Timor, Bangladesh, Tajikistan, Syria, Iraq, EI Salvador, Guyana, Zimbabwe, Zambia, equatorial guinea, Angola, Papua New Guinea, Kyrgyzstan, Cameroon, Ghana, Cambodia, Laos, Myanmar, Morocco, Kenya, Namibia, Nepal, Vanuatu
Low	Ethiopia, Togo, Gambia, Georgia, the republic of south Sudan, ivory coast, Liberia, Mauritania, Senegal, Sierra Leone, Tanzania, Uganda, Chad, Congo (gold), Djibouti, guinea, Burundi, Rwanda, Kenya, Madagascar, Mozambique, Somalia, Afghanistan, Nigeria, Sudan, Yemen

**Table 2:** The distribution of standardized incidence and mortality and DALYs of cervical cancer in different HDI levels

HDI level	Standardized incidence		Standardized mortality		DALYs	
	Median	95% CI	Median	95% CI	Median	95% CI
Upper high	10.40	6.90–12.30	3.95	2.40–5.00	216.90	123.70–263.40
High	14.30	9.20–17.90	6.50a	4.60–9.80	293.60a	232.60–361.10
Medium	16.70a	13.10–29.50	9.30a	6.40–17.00	280.80	221.00–364.50
Low	28.90ab	20.20–41.20	18.90ab	15.20–27.8	373.80a	321.70–422.50

The difference was statistically significant comparing with a and ultrahigh-HDI level group and b compared with the high-HDI level group, the difference was statistically significant

**Fig. 1:**
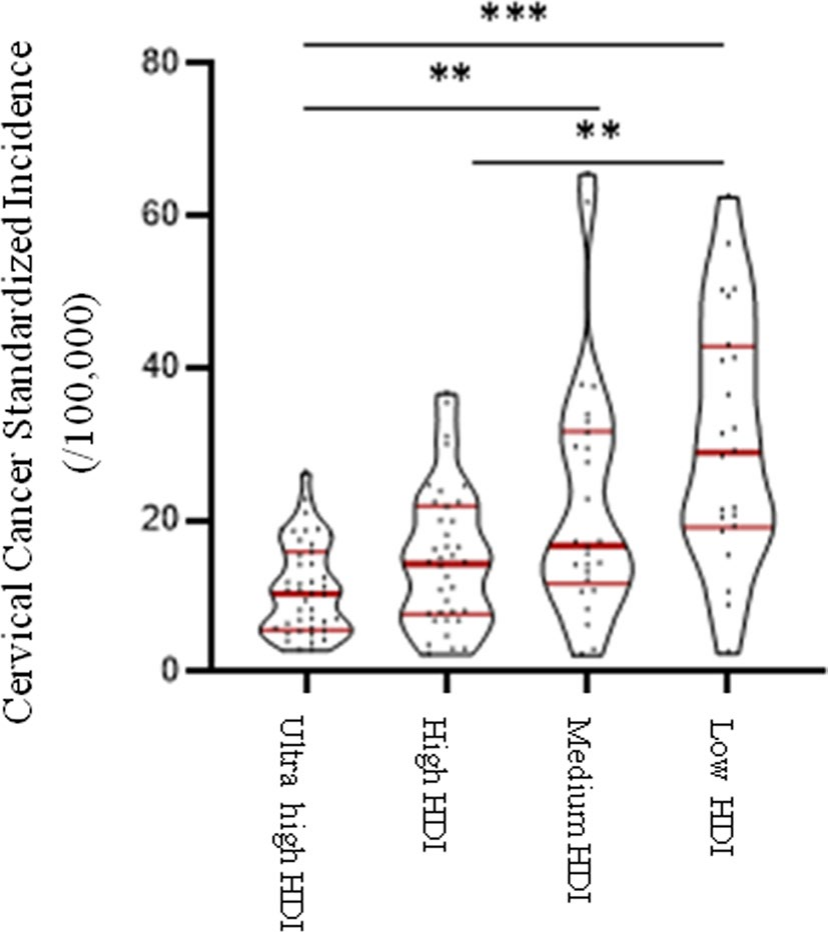
Standardized incidence of cervical cancer (/100,000)

In Fig. 1, the median standardized incidence of cervical cancer in ultrahigh-HDI countries was the lowest compared with other HDI groups, and was lower than the world average standardized incidence of 7.3/100,000. The median standardized incidence of cervical cancer in high-HDI group and medium-HDI group were similar, which were 14.3/100 000 and 16.7/100 000, respectively. The median standardized incidence of cervical cancer in low-HDI group was nearly three times higher than that in ultrahigh-HDI countries and twice as high as that in high-HDI group. Kruskal–Wallis test results showed that the standardized incidence of cervical cancer in different HDI levels was significantly different between groups (*P < *0.001). Pairwise comparison results showed that the standardized incidence of cervical cancer in ultrahigh-HDI level group was lower than that in medium-HDI level and low-HDI level groups, and the difference was statistically significant (*P < *0.01, *P < *0.001). The standardized incidence of cervical cancer was significantly different between the high-HDI level group and the low-HDI level group (*P < *0.01).

**Fig. 2:**
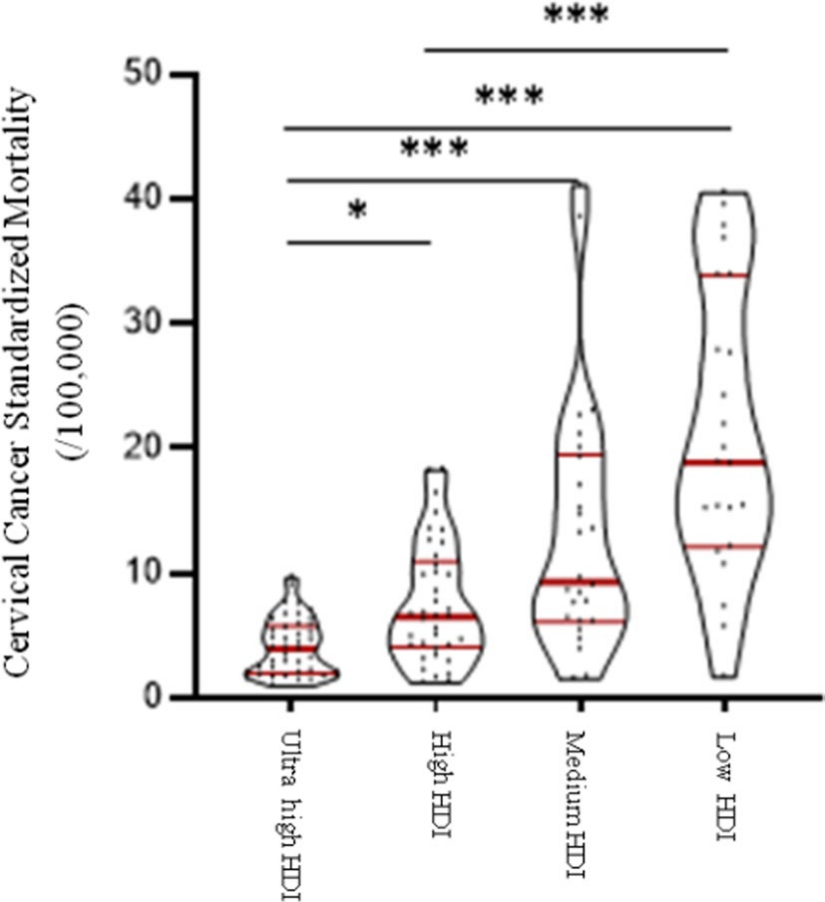
Standardized mortality of cervical cancer (/100,000)

Figure 2 shows that countries in the ultrahigh-HDI level had the lowest median standardized mortality rate for cervical cancer, with 95% confidence intervals below the world average standardized mortality rate. The median standardized mortality in the high-HDI group was lower than the world average. The standardized mortality rate of cervical cancer in the medium- and low-HDI groups were higher than the world average level, and the standardized mortality rate of cervical cancer in the low-HDI group was the highest. Kruskal–Wallis test displayed that the standardized mortality of cervical cancer in different HDI levels was statistically significant (*P < *0.001). Pairwise comparison showed that: the standardized mortality of cervical cancer in ultrahigh-HDI level group was lower than that in high-HDI, medium-HDI and low-HDI level groups, and the difference was statistically significant (*P < *0.05, *P < *0.001, *P < *0.001). The standardized mortality rate of cervical cancer was significantly different between the high-HDI level group and the low-HDI level group (*P < *0.001).

**Fig. 3:**
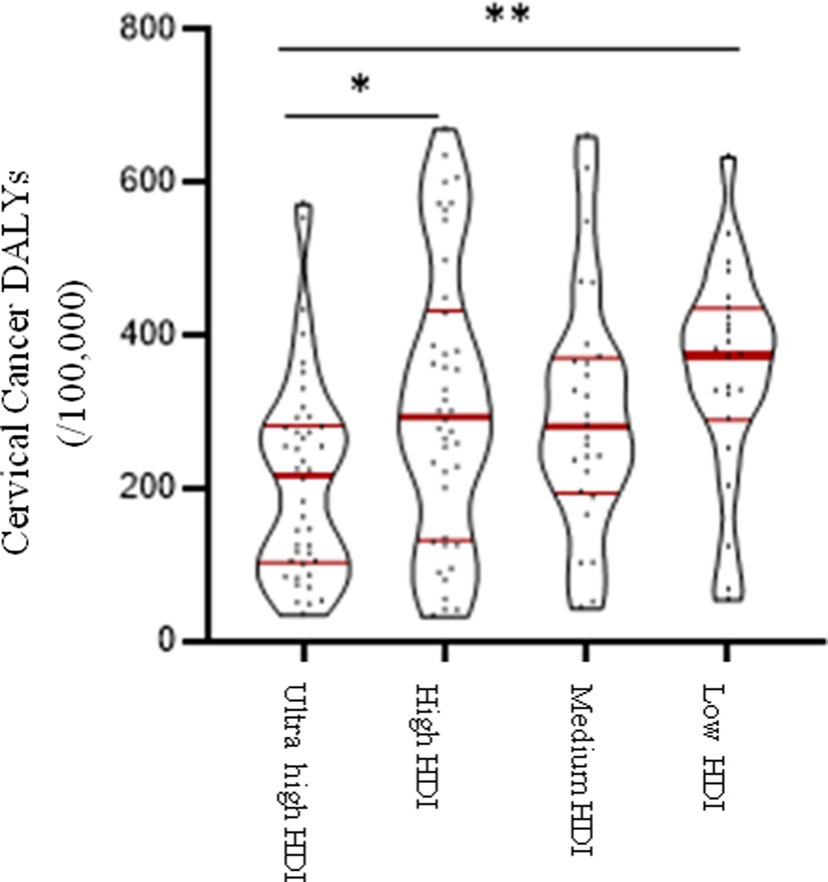
DALY distribution of cervical cancer (/100,000). The red line in the violin chart represents the quartile (thick line is the median, thin line is the upper quartile, lower quartile) *P < 0.05, **P < 0.01, ***P < 0.001 The thick line represents the concentration of most countries and the thin line represents the level of a few countries

The disability-adjusted life years (DALYs) of the low-HDI group reached 373.8/100 000, which was higher than that of the other three HDI groups. The median DALYs of high-HDI and medium-HDI group were close, and the ultrahigh-HDI group had the lowest DALYs. Kruskal–Wallis test results showed that the DALYs of cervical cancer in different HDI group levels were significantly different between groups (*P < *0.01). Pairwise comparison results showed that: compared with the ultrahigh-HDI level group, the DALYs of cervical cancer in the high-HDI level group and the low-HDI level group were statistically significant (*P < *0.05, *P < *0.01) (Fig. 3).

### Socioeconomic Influence Factors on Cervical Cancer Burden

Statistical Product and Service Solutions (SPSS) was used to analyze the correlation between and within 9 variables (Table 3). The dependent variable MIR, associated with high degree of the independent variable including “proportion of population using safely-managed sanitation services (%) (service in Table 3)”, “proportion of ever-partnered women and girls aged 15–49 years subjected to physical and/or sexual violence by a current or former intimate partner in the previous 12 months (%) (partner in Table 3)” and inequity-adjusted HDI (HDI in Table 3), and the absolute values of correlation coefficients were 0.6778, 0.5474 and 0.8032, respectively. Among them, MIR has the highest degree of association with inequity-adjusted HDI, and it is negative. The HDI value is between 0–1, and the higher the index is, the higher the degree of social and economic development, so the smaller the corresponding cervical cancer MIR is. “Service” reflects the coverage degree of safe management of environmental health services. The higher the proportion, the higher the coverage degree of environmental health services, the lower the cervical cancer MIR should be. The “partner” is positively related to MIR, the women and girls aged 15–49 years who have experienced physical and/or sexual violence by a current or former intimate partner in the past 12 months may increase their likelihood of being cervical cancer, so the greater this indicator, the higher the cervical cancer MIR in these countries.

**Table 3:** Correlation analysis of variables

Variables	1	2	3	4	5	6	7	8	9	10
1 MIR	1									
2 Population	− 0.0331	1								
3 PM2.5	0.0504	0.4501	1							
4 Coordinated	0.1277	0.2987	0.7046	1						
5 Service	− 0.6778	− 0.0520	0.1933	0.2949	1					
6 Partner	0.5474	− 0.1316	0.0138	0.0803	− 0.4316	1				
7 Ability	− 0.4926	0.1272	0.4317	0.0347	0.2015	− 0.2839	1			
8 Medical	− 0.4653	− 0.1589	− 0.1388	− 0.1049	0.5573	− 0.5347	0.4863	1		
9 Pharmacist	− 0.1263	− 0.0596	0.1359	0.5990	0.7507	0.0242	− 0.1773	0.3097	1	
10 HDI	− 0.8032	− 0.1681	− 0.1152	− 0.1859	0.7189	− 0.5731	0.5106	0.8554	0.3004	1

Overall, the annual mean concentrations of fine particulate matter (PM2.5) in urban areas (µg/m^3^) (PM2.5 in Table 3), amount of water- and sanitation-related official development assistance that is part of a government-coordinated spending plan (current US$ millions) (coordinated in Table 3), and service have positive correlationships with MIR. Other independent variables are negatively correlated with MIR. This is consistent with the actual situation, indicating that the analysis results of this study are relatively scientific and objective.

In this study, the multi-regression analysis was used to explore the influence of independent variables on dependent variables. The regression analysis results are shown in Table 4. Among them, only “average of 13 International Health Regulations core capacity scores (ability in Table 3)” is insignificant, while the remaining eight independent variables are all significant. “PM2.5”, “service”, “coordinated”, “pharmacist” and “HDI” have more significant correlationship with MIR. Among them, only “PM2.5” had a significant positive impact on cervical cancer MIR, while other significant independent variables had a significant negative impact on cervical cancer MIR. This is consistent with the results of correlation analysis, indicating that the results of this study have high reliability and validity.

**Table 4:** The regression analysis results

Variables	Regression results	Significance
Population	− 7.89e-07(1.39e–08)	0.01<*p*<0.05
PM2.5	0.0051312(0.0000581)	*p*<0.01
Coordinated	− 0.0010362(0.0000121)	*p*<0.01
Service	− 0.0090261(0.0000834)	*p*<0.01
Partner	0.0076398(0.0001243)	0.01<*p*<0.05
Ability	− 0.0001352 (0.0000432)	0.05<*p*<0.1
Medical	− 0.0013178(0.0000301)	0.01<*p*<0.05
Pharmacist	− 0.0421007(0.0004279)	*p*<0.01
HDI	− 0.6463722(0.008449)	*p*<0.01
C	1.191999(0.0036359)	*p*<0.01
R2	0.9999	
F-Value	11,214.17	

At the significance level of 5%, “total population” and “density of medical staff” had a significant negative impact on cervical cancer MIR, that is, the more total population and the greater density of medical staff, the smaller cervical cancer MIR. “The women and girls aged 15–49 years who have experienced physical and/or sexual violence by a current or former intimate partner in the past 12 months may increase their likelihood of being cervical cancer” has a significant positive effect on cervical cancer MIR, that is, the higher the index proportion, the greater the cervical cancer MIR.

Through quantitative analysis of cervical cancer mortality (MIR) and human development index (HDI) in 127 countries, this section found that a country's “amount of water- and sanitation-related official development assistance that is part of a government-coordinated spending plan”, “fine particulate matter” concentration in urban areas, “per capita net ODA for medical research and primary health sector by recipient country” had a significant positive impact on cervical cancer MIR. The density of pharmacists, the density of medical staff, the average score of 13 IHR core competencies, the proportion of population using safely managed environmental health services, the adjusted HDI index, and the total population had significant negative effects on cervical cancer MIR.

## Discussion

Few diseases reflect global inequality like cervical cancer. Cervical cancer in low- and middle-income countries have almost twice the incidence and three times the mortality rates of high-income countries. Due to the differences in cultural and economic development of countries in HDI groups, uneven development has become an overall problem affecting the healthy development of all countries. Countries with higher HDI were found to have lower prevalence and mortality of cervical cancer, vice versa. For example, 90% of African countries are low and medium HDI countries, with high prevalence of cervical cancer, and countries such as Zambia, Tanzania and Zimbabwe have morbidity and mortality rates even five times higher than the world's standardized average. On the contrary, European countries are mostly countries with ultrahigh-HDI levels and have relatively low case fatality rates. Although Montenegro has the highest incidence and mortality of cervical cancer in Europe, compared with low-HDI countries in Africa, the incidence is less than twice the world average and the mortality rate is only slightly higher than the world average. Comparing with developed countries, low health investment, weak health system foundation, lack of health care awareness and low life expectancy of residents are the main existing problems in the health system of countries in low and middle income countries. Data analysis showed that the level of social and economic development of a country was significantly correlated with the cervical cancer burden and its epidemic trend, which could be attributed to the formulation and implementation of national cancer prevention and control policies.

WHO Global Action Plan for the Prevention and Control of Noncommunicable Diseases 2013–2020 cited HPV vaccination and cervical cancer screening and treatment as the most worthwhile cervical cancer prevention and control interventions [7]. This is specified in each country's strategy or plan. The cancer prevention and control policies in countries with high HDI have achieved relatively ideal implementation effects. For example, in New Zealand, an ultrahigh-HDI country, the incidence of cervical cancer decreased by 56% between 2009 and 2013 compared with 20 years ago, following the implementation of the 2005–2010 New Zealand Cancer Prevention and Control Strategy. In 2018, HPV vaccination coverage for girls aged 15 years in New Zealand reached 68%. Over the past 20 years, 300,000 women in New Zealand have now been vaccinated against HPV, leading to a steady decline in cervical cancer incidence and mortality in New Zealand [8]. In Slovenia, the National Cancer Prevention and Control Plan 2010–2015 has carried out cervical cancer screening program throughout the country. The incidence of cervical cancer has been decreasing year by year for 5 years, and the 5-year survival rate after surgery has exceeded 80% [9]. In addition, the allocation of health resources in countries with high HDI is relatively average, which effectively controls the incidence of cervical cancer. For example, the Malaysian Ministry of Health requires that professional doctors engaged in gynecological internal and surgical treatment should have 1–2 years of overseas study experience. After 2017, each cancer center is required to have at least two professional gynecologists for daily examination [10].

However, for countries with relatively backward social and economic development level, cancer prevention and control policies have been formulated, but it is difficult to implement and little effect. The main existing problems are:

### Low HPV Vaccination Coverage

The Polish Ministry of Health has not included the HPV vaccine in the free national immunization programme due to limited national health funding, so it is only available to women of school age in areas funded by local governments. In 2010, only 30,000 school-age girls in Poland were given the nine-valent HPV vaccine free of charge through bricks-to-mortar funding [11]. Nigeria, Kenya, Cameroon, Ethiopia and other African countries cannot afford the high cost of the HPV vaccine to give it to all eligible people for free [12–15]. Ethiopia is relying on funding from the Global Alliance for Vaccines and Immunization (GAVI) to provide unpaid vaccinations to school-age girls from 2016 to 2020 [15]. The promotion of HPV vaccine in China is faced with the problem of high price and short supply. According to statistics, about 9.74 million women completed HPV vaccination from 2017 to 2020, and the vaccination rate of women aged 9–45 was only about 3% [16]. The promotion and popularization of HPV vaccine in Saudi Arabia, Qatar and other countries in the Middle East are greatly influenced by cultural background factors such as religious belief, which leads to the difficulty of improving vaccination coverage [17].

### Poor Regional Health Resource Allocation

The way a country implements cervical cancer screening depends on the country's investment in public health, which directly affects the allocation efficiency of medical and health resources. At present, it is widely accepted that the main screening methods for cervical cancer are visual inspection, colposcopy, cytology and HPV detection [18]. Among them, due to the low cost of visual observation and colposcopy, it has become the main method for cervical cancer screening in countries with low socioeconomic development level, such as Africa. More economically developed countries use more expensive and accurate cytology and DNA HPV testing. In addition, there are differences in the allocation of health resources between urban and rural areas. For example, HPV vaccination services in China are mainly provided in urban health services, while vaccination and cervical cancer screening are not available in rural area level health centers [19]. In Kenya, Cameroon, Nigeria and other African countries, the medical institutions are mainly distributed in the capital or big cities, and the rural areas cannot provide gynecological examination and other medical services, which makes the prevention and treatment of cervical cancer difficult.

### Weak Public Education Awareness

In 2009, Zimbabwe established a National Cancer Prevention and Control Committee and introduced the HPV vaccine to reduce the incidence and mortality of cervical cancer. However, due to the lack of cervical cancer prevention awareness among women, more than 35% of Zimbabwean women expressed reluctance to be vaccinated [20]. Statistics from Mongolia show that 88% of cervical cancer cases are advanced when detected. This is mainly due to the lack of awareness of cervical cancer prevention and the symptoms of the disease among women, and the difference in medical resources allocation between rural and urban areas [21]. It is not uncommon for women to have no one to save and no medicine to treat. In 2012, about 36% of the target population in Poland participated in the cervical cancer screening programme, and the results show that women living in large cities are much more aware of screening than women in small and medium-sized cities and rural areas, and the participation rate of women with low education is lower [11].

Given the current prevalence status of cervical cancer in the world, WHO released the Global Strategy to Accelerate the Elimination of Cervical Cancer in 2020, which aimed to reduce the cervical cancer incidence to 4 per 100 000 in every country by 2030, as a global public health issue [22]. To achieve this goal, WHO is actively promoting a range of interventions across the world, including human papillomavirus vaccination (HPV), precancerous screening and treatment, and the improvement and popularization of information systems for cancer management. For cervical cancer prevention and control policies to be most effective, cervical cancer prevention and control and interventions must be integrated into national strategies and implemented in health service platforms that take into account women's needs, women's social context, and personal, cultural, social, institutional and economic barriers to women's access to health services. It is essential to implement people-oriented comprehensive health services that respect and protect women’s rights.

Above all, this study systematically and innovatively investigates the current status of cervical cancer burden and its influence factors in 127 countries with different HDI category levels in terms of epidemiology, health economic, and policy effectiveness analysis. In essence, the differences of cervical cancer control in each country depends on the policy implementation effect. Countries with high HDI has sound public health infrastructure to implement the cancer control strategy to the real case. However, countries with low HDI have poor health resource allocation and inaccessible policy intervention. This study provides evidence-based data analysis and outcome to realize the prevalence of cervical cancer burden in 127 countries and further promotes the national cancer control policy improvement so as to achieve the goal of cervical cancer elimination and health equity in the world ultimately.

## Conclusion

The statistical and quantitative analysis showed that the cervical cancer burden is varies in different HDI countries. The high the HDI is, the low the prevalence of cervical cancer. Also, the government coordinated spending, air and water pollution, and intimate partners and other socioeconomic factors have positive impact on cervical cancer burden. The cancer control experiences of high- and ultrahigh-HDI countries indicated that effective implementation of cancer policies can reduce the cervical cancer disease burden. The essential reason why cervical cancer burden is heavier in low and medium HDI country because the cancer control strategy implementation is little effective. We found that low HPV vaccination coverage, poor regional health resource allocation, and weak public education awareness are the main existing problems in lower HDI countries. Therefore, the research suggests that cervical cancer control plan should be a part of national strategy and focus on population-based HPV vaccination, large-scale screening, public health education for all, and increase the financial investments, lower disparities and inequalities among different social status women groups, and strengthen the political supports both from social and economic sides.

## Data Availability

The data are available from open access databases, such as WHO tomorrow cancer registry system, global burden disease database, and Human Development Report.

## Abbreviations

HPV: Human papilloma virus
HDI: Human Development Index
GNI: Gross national income
MIR: Mortality-to-incidence ratio
DALY: Disability-adjusted life years
GBD: Global burden of disease database
SPSS: Statistical product and service solutions
GAVI: Global Alliance for Vaccines and Immunization
WHO: World Health Organization
