# Correction: The Study on Cervical Cancer Burden in 127 Countries and Its Socioeconomic Influence Factors

**DOI:** 10.1007/s44197-023-00087-3

**Published:** 2023-01-24

**Authors:** Tingting Xu, Xueling Yang, Xiaoning He, Jing Wu

**Affiliations:** 1 School of Pharmaceutical Science and Technology, Tianjin University, Tianjin, 300072 China; 2 Tianjin Medical University Cancer Institute and Hospital, Tianjin, 300060 China

**Correction: Journal of Epidemiology and Global Health** 10.1007/s44197-022-00081-1

In Table 3, the column heading of the first column contained the Chinese word for "Variables" and was corrected to English in the following Table 3.

**Table 3:** Correlation analysis of variables

Variables	1	2	3	4	5	6	7	8	9	10
1 MIR	1									
2 Population	− 0.0331	1								
3 PM2.5	0.0504	0.4501	1							
4 Coordinated	0.1277	0.2987	0.7046	1						
5 Service	− 0.6778	− 0.0520	0.1933	0.2949	1					
6 Partner	0.5474	− 0.1316	0.0138	0.0803	− 0.4316	1				
7 Ability	− 0.4926	0.1272	0.4317	0.0347	0.2015	− 0.2839	1			
8 Medical	− 0.4653	− 0.1589	− 0.1388	− 0.1049	0.5573	− 0.5347	0.4863	1		
9 Pharmacist	− 0.1263	− 0.0596	0.1359	0.5990	0.7507	0.0242	− 0.1773	0.3097	1	
10 HDI	− 0.8032	− 0.1681	− 0.1152	− 0.1859	0.7189	− 0.5731	0.5106	0.8554	0.3004	1

The original article has been corrected.

